# Effects of grape pomace and seed polyphenol extracts on the recovery of gut microbiota after antibiotic treatment in high‐fat diet‐fed mice

**DOI:** 10.1002/fsn3.1141

**Published:** 2019-08-11

**Authors:** Feng Lu, Fengjiao Liu, Qian Zhou, Xiaosong Hu, Yan Zhang

**Affiliations:** ^1^ College of Food Science and Nutritional Engineering China Agricultural University Beijing China; ^2^ National Engineering Research Center for Fruits and Vegetables Processing Ministry of Science and Technology Beijing China

**Keywords:** antibiotics, grape pomace polyphenol extracts, grape seed polyphenol extracts, gut microbiota, high‐fat diet

## Abstract

The widespread use of antibiotics all over the world increases the risk of many metabolic diseases by altering the gut microbiota. Grape by‐products are of particular interest in the prevention of metabolic diseases, while only minimum amounts of these wastes are up‐graded or recycled at present. The study investigated the effect of grape pomace (GPE) and seed (GSE) polyphenol extracts on the recovery of gut microbiota after antibiotic cocktail treatment in high‐fat diet‐fed (HFD) mice. C57BL/6J mice were fed HFD together with antibiotic treatment by drinking water for 3 weeks. Then, GPE and GSE were supplemented for 7 days after withdrawal of antibiotics. The gut microbiota was characterized by a significant loss of diversity and certain important taxon after a 3‐week antibiotic cocktail treatment. The GPE and GSE supplementation during the restore period of gut microbiota had some positive effects. The relative abundance of gut microbiota was improved by GPE and GSE compared to the spontaneous recovery group. And gut microbiota diversity was also greatly changed by GPE and GSE, being indicated by the changes of *Verrucomicrobia* and *Akkermansia* in feces. These findings suggested that grape polyphenol extracts have a great influence on the recovery of gut microbiota after antibiotics and high‐fat diet treatment.

## 1 INTRODUCTION

Human gut is populated by a vast number of bacterial species (more than 800) that reach the highest concentrations in the colon (up to 1,012 cells per gram of feces), which helps to develop important metabolic and immune functions, with a marked effect on the nutritional and health status of the host (Cardona, Andrés‐Lacueva, Tulipani, Tinahones, & Queipo‐Ortuño, 2013; Laparra & Sanz, 2010). The composition and diversity of the gut microbiota vary markedly across individuals and are easy to be affected by diets, medicine, diseases, especially antibiotics which are ubiquitous in modern life. Notably, as the survey conducted by Mikkelsen, Allin, and Knop (2016) that the widespread use of antibiotics all over the world increases the risk of many metabolic diseases such as obesity and type 2 diabetes. Also, most of these damages to health were caused by the negative effects of antibiotics on the gut microbiota, including the long‐term dysbiosis of the microbial ecology, low microbial biodiversity, increasing of pathogenic bacterium, and decreasing of beneficial bacterium (Zarrinpar, Chaix, Yooseph, & Panda, 2014), moreover, reducing of functional diversity (Lange, Buerger, Stallmach, & Bruns, 2016).

Thus, it seems to be very important to find out that how to partly reverse the negative effects of antibiotics on gut microbiota (Dethlefsen & Relman, 2011; Suárez‐Zamorano et al., 2015). Growing evidence supported that grape polyphenol extracts may play beneficial roles on the physiological health of human due to its interaction with microbiota (Milenkovic, Jude, & Morand, 2013; Del Rio et al., 2013). Dietary polyphenols as the parent compounds or their metabolites pass to the colon where they are degraded by the action of the local microbiota, giving rise principally to small phenolic acid and aromatic catabolites that are absorbed into the circulatory system (Cardona et al., 2013; Choy et al., 2014; Lee, Jenner, Low, & Lee, 2006; Monagas et al., 2010; Del Rio et al., 2013; Tenore, Campiglia, Ritieni, & Novellino, 2013). Meanwhile, polyphenols and their metabolites may also modify the composition, metabolism, or activity of gut microbiota, further affect intestinal ecology (Kim et al., 2015; Laparra & Sanz, 2010). There were a few studies on the impact of dietary polyphenols on the human gut microbiota. These researches showed that polyphenols can significantly modulate the growth of selected gut microbiota in humans. For instance, the polyphenol‐rich cranberry extract downregulated the *Firmicutes* to *Bacteroidetes* ratio and expanded the *Akkermansia muciniphila*, decreased *Barnesiella* spp. Proanthocyanidin‐rich red wine extracts shifted bacteria composition of rats from a predominance of *Bacteroides, Clostridium,* and *Propionibacterium* spp. to a predominance of *Bacteroides, Lactobacillus,* and *Bifidobacterium* spp. Proanthocyanidin‐rich extract from grape seeds given to healthy adults for 2 weeks was able to significantly increase the number of *Bifidobacterium* (Dolara et al., 2005; Kim et al., 2015; Queipo‐Ortuño et al., 2012; Rastmanesh, 2011; Tzounis et al., 2008). However, the concentration on the recovery effect of polyphenol on gut microbiota, after antibiotics damages, remains quite limited.

Thus, the main goal of this work was to investigate the effects of grape pomace polyphenol extracts (GPE) and grape seed polyphenol extracts (GSE) on the recovery of gut microbiota after antibiotic cocktails treatment in HFD mice so that elaborate the potential beneficial effects of polyphenols on gut microbiota.

## 2 MATERIALS AND METHODS

### 2.1 Extracts of polyphenols from grape pomaces (GPE)

Extracts of polyphenols used in the investigation were extracted from the Kyoho Grape (*Vitis vinifera“Kyoho”*) pomace according to the methods of Ghafoor, Choi, Jeon, and Jo (2009), with some modifications. Briefly, the powdered pomace was extracted by ultrasound assist procedure using acidified ethanol water (50%) media for 25 min according to our previous work. After centrifugation (4°C, 12,000 *g*, 10 min) and concentration under vacuum conditions at 40°C, the crude polyphenol extracts were then purified by the macroporous adsorption resin AB‐8, and the effluents were concentrated after desorption. At last, the purified grape polyphenol extracts (GPE) were freeze‐dried using a lyophilizator (LGJ‐12, Beijing Songyuan Huaxing Technology Develop Co., Ltd.) then stored at −80°C until using. And 2.812 g grape pomace polyphenol (GPE) could be extracted from 100 g grape pomace. The commercial dry grape seed extracts (GSE) were provided by Tianjin Jianfeng Natural (Tianjin Jianfeng Natural R&D Co., Ltd.).

**Figure 1:**
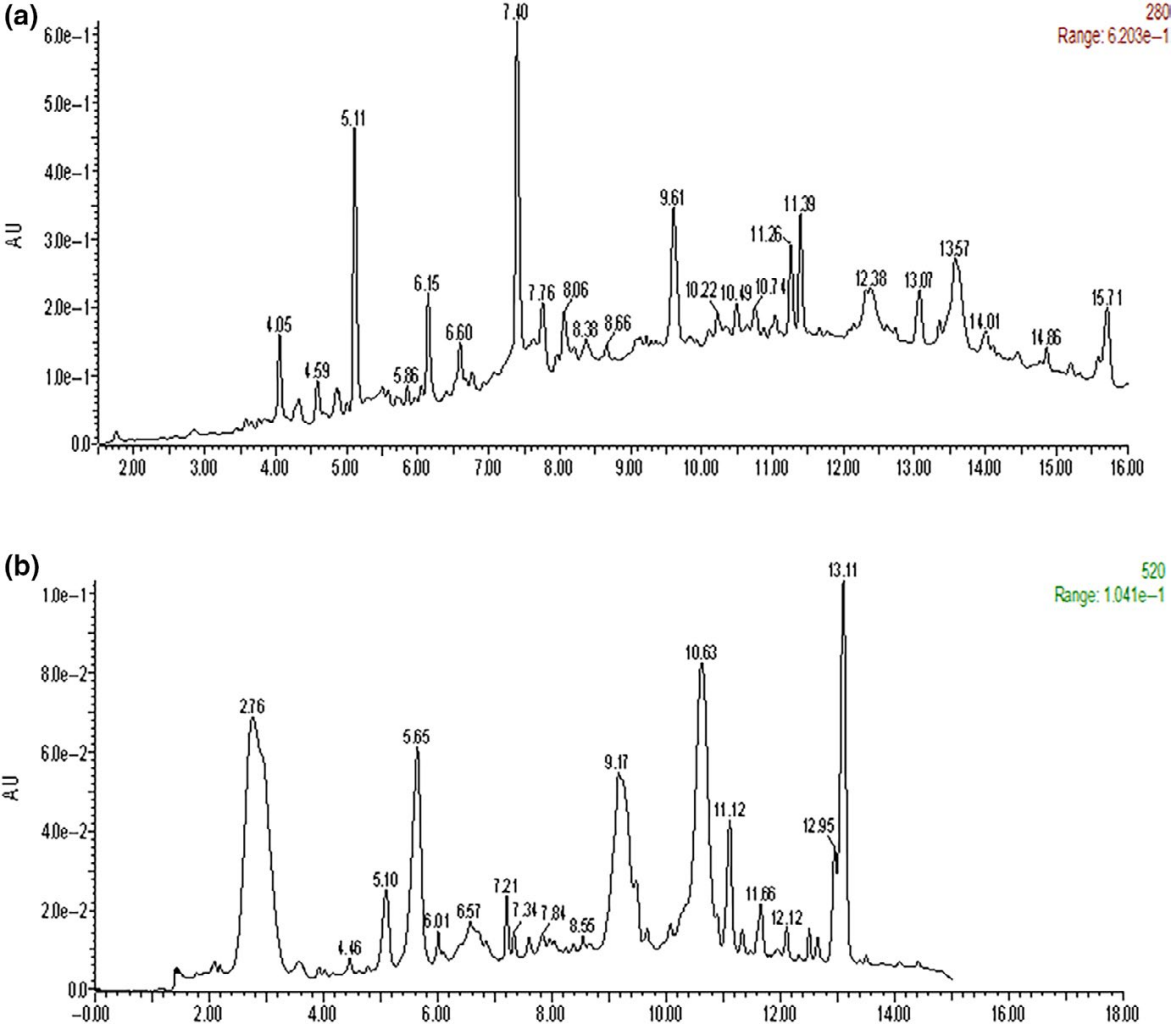
UPLC/MS profile of grape pomace extracts detected at (a) 280 nm for phenolic acid, (b) 520 nm for anthocyanins

The UV was used to determine total polyphenol content and total proanthocyanidin content of GPE and GSE. Total polyphenol content of GPE and GSE, expressed as of equivalent gallic acid (mg of gallic acid equivalents (GAE)/g extract), was 80.60 g GAE/100 g and 86.33 g GAE/100 g, respectively. Proanthocyanidins, the main polyphenolic components in grape pomace, was expressed as equivalent catechin, namely, mg of catechin equivalents (CAT)/g extract. The contents were 63.47 g CAT/100 g and 91.00 g CAT/100 g in GPE and GSE, respectively. The anthocyanin and phenolic profile determined by UPLC‐MS is shown in Table 1 and Figure 1, where the specific types of proanthocyanidins of GPE were showed as proanthocyanidins dimer, galloyl proanthocyanidin dimer, and proanthocyanidins trimer (Table 1). Among total proanthocyanidins, oligomeric proanthocyanidin accounts for 63.14% and proanthocyanidin B2 accounts for 1.84%.

**Table 1:** Composition of grape pomace extracts

Polyphenols	tR (min)	MS (m/z)	MS/MS (m/z)	λ max
Anthocyanin (ESI^+^)				
Peonidin‐3‐caffeoyl glucoside	2.76	625^+^	301	518.05
Malvidin‐3‐caffeoyl glucoside	2.96	655^+^	331	522.05
Pelargonidin‐3‐coumarin glucoside	4.46	579^+^	271	525.05
Peonidin‐3‐glucoside	5.10	463^+^	301	517.05
Malvidin‐3‐glucoside	5.65	493^+^	331	527.05
Delphinidin‐3‐glucoside	7.60	465^+^	303	526.05
Malvidin‐3‐caffeoyl diglucoside	9.17	817^+^	655/331	530.05
Malvidin‐3‐coumarin diglucoside	9.50	801^+^	639/331	535.05
Petunidin‐3‐caffeoyl glucoside	9.67	641^+^	317	530.05
Malvidin‐3‐caffeoyl glucoside	11.12	655^+^	331	531.05
Pelargonidin‐3‐caffeoyl glucoside	11.32	595^+^	475	525.05
Petunidin‐3‐coumarin glucoside	11.66	625^+^	317	530.05
Malvidin‐3‐coumarin glucoside	12.12	639^+^	‐‐	525.05
Peonidin‐3‐coumarin glucoside	12.95	609^+^	301	526.05
Malvidin‐3‐coumarin glucoside	13.11	639^+^	331	534.05
Phenolic (ESI^−^)				
Proanthocyanidin trimer	1.75	865		273.05
Single glucogallin	2.38	331		254.05
Proanthocyanidins dimer	4.05	577		279.05
Proanthocyanidins trimer	4.59	865/577		279.05
Proanthocyanidins trimer	4.89	865/577		279.05
Catechinic acid	5.11	289		279.05
Proanthocyanidins dimer	5.37	577		279.05
Proanthocyanidins trimer	5.51	865/577/289		279.05
Proanthocyanidins trimer	5.59	865/577		280.05
Proanthocyanidins trimer	6.15	865/577		279.05
Proanthocyanidins trimer	6.74	865/577		279.05
Epicatechin	7.40	289		279.05
Proanthocyanidins trimer	7.76	865/289		278.05
Galloyl proanthocyanidins dimer	8.06	729		279.05
Proanthocyanidins trimer	8.25	865/577		279.05
Proanthocyanidins trimer	8.38	865		278.05
Isorhamnose‐3‐O‐glucuronide	8.66	491		279.05
Proanthocyanidins trimer	10.22	865		279.05
Epigallocatechin gallate	10.49	441		279.05
Tannic acid	10.74	817		280.05
Proanthocyanidins trimer	11.01	865/577/289		278.05
Isorhamnose‐3‐O‐hexose	11.26	477		351.05
Quercetin‐3‐O‐glucoside	11.39	463		352.05
Quercetin‐3‐O‐rhamnoside	13.07	447		344.05
Isorhamnose‐3‐O‐hexose	13.57	477		272.05
Dimethoxyquercetin‐O‐coumarin Hexoside	14.01	653		280.05

### 2.2 Animals and experimental design

All animal experimental procedures were performed and approved by the Ethical Committee of Peking University, Health Science Center (Beijing, China) with mice housed in specific pathogen‐free (SPF) conditions. Male C57BL/6J mice at the age of 6 weeks were purchased from Vital River Laboratories and were housed under the standard laboratory conditions (22 ± 2°C, 55 ± 5% relative humidity, a 12‐hr light/dark cycle) with free access to food and drinking water. Mice acclimatized on water and standard chow diet ad libitum for 1 week prior to the initiation of the experiment. All efforts were made to minimize animal suffering.

Acclimatized animals were weighed and randomly divided into five groups of 10 animals each and kept five per cage. One group of animals were normally raised with normal chow (NC) diet containing 10% kcal from fat (D12450B, Research Diets Inc.), without antibiotics as healthy controls (namely, NC + Abx^−^ group). Four experiment groups were given a high‐fat diet containing 60% Kcal from fat (D12492, Research Diets Inc.) during the experiment process and were initially given a 3‐week treatment of the following antibiotics in their drinking water (0.5 g/L of vancomycin, 1 g/L of ampicillin, 1 g/L of neomycin sulfate, 1 g/L of metronidazole), as previously described (Suez et al., 2014) with some modifications. And the diet composition is exhibited in Table 2 according to the information supplied by manufacturer. Antibiotics were supplied every 2 days. One of the experiment groups was only received antibiotics for 3 weeks, without recovery (namely, HFD + Abx^+^ group). While other three groups, after 3‐week antibiotic treatment, have been immediately repopulated with microbiota for 7 days after withdrawal of antibiotics, meanwhile these three groups additionally being received by daily gavage either normal saline (namely, HFD + Abx^+/−^ group) or a solution of 200 mg/kg·bw of GPE in normal saline (namely, HFD + Abx^+/−^ + GPE group) and GSE (namely, HFD + Abx^+/−^ + GSE group) in normal saline. Body weight of each mouse was recorded weekly, and the volume of solutions to force feed was adjusted according to the weight of mice. Feces were collected from all mice of five groups in the last two consecutive days. Stool samples were snap‐frozen in liquid nitrogen before storage at −80°C until using.

**Table 2:** The composition of normal and high‐fat diet

Class description	Ingredients (g)	Diets	
		ND	HFD
Protein	Casein, Lactic, 30 Mesh	200.00	200.00
	Cystine, L	3.00	3.00
Carbohydrate	Sucrose, Fine Granulated	354.00	72.80
	Starch, Corn	315.00	–
	Lodex 10	35.00	125.00
Fiber	Solka Floc, FCC200	50.00	50.00
Fat (plant source)	Soybean Oil, USP	25.00	25.00
Fat (animal source)	Lard	20.00	245.00
Mineral	S10026B	50.00	50.00
Vitamin	Choline Bitartrate	2.00	2.00
	V10001C	1.00	1.00
Dye	Dye	0.05	0.05
Total	Total	1,055.05	773.85

### 2.3 Biochemical analysis of serum lipid

For analysis of serum lipid, a 3,100 automatic biochemistry analyzer (Hitachi Ltd.) was used to determine triglyceride (TG), total cholesterol (TC), low‐density lipoprotein (LDL), and high‐density lipoprotein (HDL) content in serum.

### 2.4 Extraction of total genomic DNA

Total DNA was extracted from the fecal samples using a QIANamp Fast DNA Stool mini kit (Qiagen) according to the manufacturer's instructions. The integrity of the extracted DNA was examined by electrophoresis in 1% (wt/vol) agarose gels. Based on the quantity and the quality of the DNA extracted, samples were selected to perform the consequent sequencing.

### 2.5 PCR amplification and sequencing analysis

Fecal DNA samples were used as the template for PCR amplification of the V4 hypervariable regions of 16S rRNA genes in a PCR system (Bio‐Rad). The V4 region is one of the commonly used regions for microbiota sequencing (Claesson et al., 2011; David et al., 2014; Lozupone et al., 2013; Lukens et al., 2014; Yatsunenko et al., 2012). Some studies have found that the results of sequencing in the V4 region and sequencing in the V4‐5 region are very close (Walters et al., 2016). That is to say, the V4 region can represent the V4‐5 region and can circumvent the problem of shorter sequence sequencing reducing quality of detection. The amplification program was 3 min of denaturation at 95°C, 27 cycles of 30 s at 95°C, 30 s for annealing at 55°C, and 45 s for elongation at 72°C, and a final extension at 72°C for 10 min using the 515F (5′‐barcode‐ACTCCTACGGGAGGCAGCAG‐3′) and 806R (5′‐GGACTACHVGGGTWTCTAAT‐3′), where barcode was an 8‐base sequence unique to each sample. PCR amplification was performed on GO Taq^®^Hot Start Colorless Master Mix System (Promega). The resulted PCR products were extracted from a 2% agarose gel and further purified using the QIAquick^®^ PCR purification Kit (Qiagen). Purified amplicons were quantified using QuantiFluor‐ST Handheld Fluorometer with UV/Blue Channels (Promega Corporation).

Sequencing of the PCR amplification products was performed on an Illumina Miseq platform (Illumina) at Tianyi Health Sciences Institute Co., Ltd. Briefly, the 16S rRNA gene sequencing data were filtered and trimmed and further classified into operational taxonomic units (OTUs) within a 0.03 difference (equivalent to 97% similarity). And chimeric sequences were identified and removed using UCHIME. The taxonomy of each 16S rRNA gene sequence was analyzed by RDP Classifier algorithm (http://rdp.cme.msu.edu/) against the silva (SSU115) 16S rRNA database (Quast et al., 2013). Refraction and alpha diversity analysis were performed using Mothur (version V.1.30.1).

### 2.6 Statistical analysis

All data were analyzed using one‐way analysis of variance (ANOVA) and expressed as mean ± *SEM* (standard error of mean). Significant differences between the means were further analyzed using the Tukey test (*p* < .05).

## 3 RESULTS

### 3.1 Effects of GPE and GSE on the recovery of serum lipid parameter

As shown in Table 3, HFD + Abx^+^ group and HFD + Abx^+/−^ group mice exhibited dyslipidemia as evidenced by significant increased levels of TC, TG, and LDL and decreased level of HDL. GSE supplementation presented a significantly decreasing effect on plasma TC in mice (*p* < .05).

**Table 3:** Effects of GPE and GSE on the serum lipid level

Groups	TC	TG	LDL‐c	HDL‐c
ND + Abx^−^	3.243 ± 0.618a	0.568 ± 0.418a	1.023 ± 0.317a	3.707 ± 0.992b
HFD + Abx^+^	3.995 ± 0.314b	0.809 ± 0.230b	1.559 ± 0.338b	3.400 ± 0.464a
HFD + Abx^+/−^	3.489 ± 0.408b	0.628 ± 0.165b	1.425 ± 0.066b	3.467 ± 0.287a
HFD + Abx^+/−^ + GPE	3.477 ± 0.252b	0.695 ± 0.139b	1.448 ± 0.085b	3.413 ± 0.223a
HFD + Abx^+/−^ + GSE	2.812 ± 0.488a	0.730 ± 0.109b	1.394 ± 0.058b	3.387 ± 0.557a

Note

The means in the same column as different letters differ from each other, *p* < .05.

### 3.2 Effects of GPE and GSE on the recovery of microbiota

#### 3.2.1 Sequencing depth and community diversity

Changing community composition was assessed in five groups (ND + Abx^−^ group, HFD + Abx^+^ group, HFD + Abx^+/−^ group, HFD + Abx^+/−^ + GPE group, HFD + Abx^+/−^ + GSE group). Good's coverage (estimated probability that the next read will belong to a refOTU that has already been found) was up to 99.6%–99.8% for individual samples (Table 4), showing that the sequencing can be representative and that the sequencing depth covered rare new phylotypes and most of the diversity.

**Table 4:** Sequencing depth and diversity of microbiota

Groups	refOTUs	Good's	ACE	Chao 1	Shannon	Simpson
ND + Abx^−^	361 ± 53a	0.996	558.42 ± 136.68a	519.55 ± 86.76a	3.69 ± 0.43a	0.95 ± 0.03c
HFD + Abx^+^	222 ± 28b	0.997	423.44 ± 66.22a	364.83 ± 42.87b	0.43 ± 0.22b	0.13 ± 0.08a
HFD + Abx^+/−^	234 ± 19b	0.998	401.22 ± 59.53a	356.38 ± 30.06b	0.86 ± 0.25b	0.35 ± 0.15b
HFD + Abx^+/−^ + GPE	244 ± 15b	0.998	462.40 ± 112.22a	392.94 ± 45.42b	1.00 ± 0.71b	0.39 ± 0.30b
HFD + Abx^+/−^ + GSE	249 ± 40b	0.997	568.70 ± 172.21a	410.45 ± 87.66b	1.03 ± 0.34b	0.54 ± 0.19b

Note

The means in the same column as different letters differ from each other, *p* < .05.

The 3‐week treatment of antibiotics did significantly reduce the richness and diversity of fecal microbiota. The refOTUs decreased in relative abundance significantly after Abx‐treatment and present an increasing tendency after withdrawal of antibiotics, as well as the same change with the abundance and diversity indexes of ACE, Chao, Shannon, and Simpson. In spite there was no significant difference among HFD + Abx^+/−^ group, HFD + Abx^+/−^ + GPE group and HFD + Abx^+/−^ + GSE group on the fecal microbiota according to Table 4. Being given GPE and GSE during the restored period, the abundance and diversity of microbiota showed increasing tendency compared to the HFD + Abx^+/−^ group. Especially, GSE intake had a greater impact. The results showed that GPE and GSE may have some positive effects on the microbiota recovery during reproduce period, and further study is needed.

**Figure 2:**
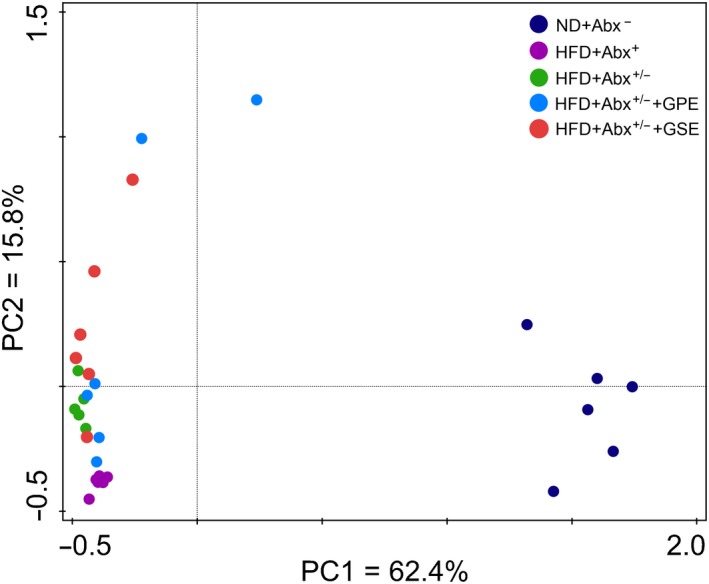
PCA analysis

UniFrac‐based principal analysis (PCA) revealed a distinct clustering of microbiota composition of ND + Abx^−^ group versus HFD + Abx^+^, HFD + Abx^+/−^ HFD + Abx^+/−^ + GPE and HFD + Abx^+/−^ + GSE group (Figure 2).

#### 3.2.2 Effects on microbiota composition

**Figure 3:**
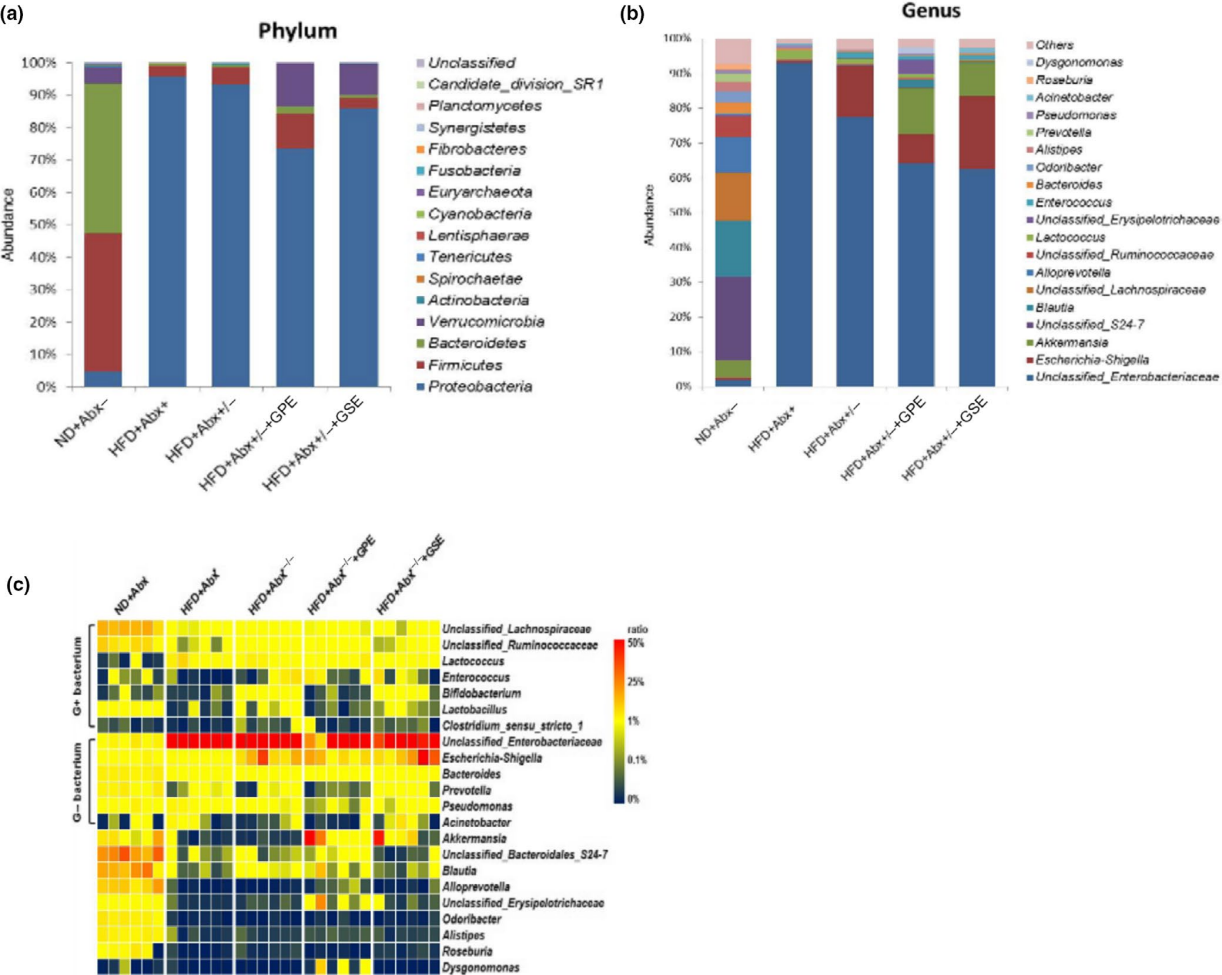
(a) Microbiota composition in mice feces of before and after antibiotic treatment and the end of resurrection with GPE and GSE supplementation. Bacterial taxonomic profiling in the phylum level. (b) Bacterial taxonomic profiling in the genus level. (c) Heatmap showing the abundance of 22 OTUs significantly altered by GPE and GSE

Two bacterial phyla dominate the gut of normal mice: *Firmicutes* (42.82%) and *Bacteroidetes* (46.16%), whereas *Proteobacteria*, *Actinobacteria*, and *Verrucomicrobia* phyla were less frequently found (Figure 3a). After antibiotic cocktail treatment, there were profound effects on fecal bacteria composition, and taxonomic richness decreased markedly (Figure 3a,b). This post‐antibiotic dysbiosis was characterized by a significantly reduced diversity of the phyla *Firmicutes*, *Bacteroidetes*, *Actinobacteria* and accompanied by an overgrowth of the phyla *Proteobacteria*. 1 week after the withdrawal of antibiotic cocktail, communities of HFD + Abx^+/−^ group began to return to their initial state, but the return was incomplete, as described in (Figure 3a,b). However, GPE and GSE supplementation during the resurrecting process of microbiota increased its composition and improved its complexity (Figure 3a,b). At phylum level, relative abundance of *Verrucomicrobia* tended to be significantly greater while relative abundance of *Actinobacteria* was markedly lowered by GPE and GSE compared to HFD + Abx^+/−^ group (Figure 3a).

Further analysis of the bacterial phyla at a genus level showed post‐antibiotic a significant loss on certain important genuses, such as *Bacteroides*, *Blautia*, *Alloprevotella, and Akkermansia* (Figure 3b). In HFD + Abx^+^ group, after 1‐week recovery, the relative abundance of some genuses has increased, for example, *Escherichia‐Shigella* and *Acinetobacter* (Figure 3b), where the return of the whole gut microbiota community was limited. While GPE supplementation and GSE supplementation during the resurrecting process of microbiota contribute to the recovery of gut microbiota, for increasing its composition and improving its complexity on the genus level (Figure 3b). According to Figure 3b and Table 5, the decreased relative abundance of *Akkermansia* in feces was markedly recovered by GPE and GSE compared to the HFD + Abx^+/−^ group (0.01%), changing from 0.0496% after treatment of antibiotic to 13.35% and 9.61%, respectively, even higher than the initial state (5.01%).

**Table 5:** GPE and GSE administration modulated the recovery of gut microbiota of antibiotic‐treated mice in genus level (%)

Groups	*Akkermansia*	*Alloprevptella*	*Prevotella*	*Streptococcus*
ND + Abx^−^	5.01282b	10.3515a	2.4263a	0.0264c
HFD + Abx^+^	0.049602c	0.0075c	0.1026c	0.0045c
HFD + Abx^+/−^	0.014881c	0.0024c	0.1142c	0.3500a
HFD + Abx^+/−^ + GPE	13.352131a	0.0388b	0.0413d	0.0067c
HFD + Abx^+/−^ + GSE	9.60679a	0.0113c	0.1731b	0.1709b

Note

The means in the same column as different letters differ from each other, *p* < .05.

GPE and GSE also increased *Alloprevotella* relative abundance greatly, changing from 0.0075%, after treatment of antibiotic, to 0.0388% and 0.0113%, respectively, compared to the HFD + Abx^+/−^ group (0.0024%). Besides, GSE significantly elevated the relative abundance of *Prevotella*, changing from 0.1026% (after treatment of antibiotic) to 0.1731%. Taking GPE and GSE not only increased microbiota abundance, but also decreased some taxon. Relative abundance of *Streptococcus* was significantly lowered by GPE and GSE intake (Table 5). Although the relative abundance of these microbiota was less than 1%, the restorative effect of 1‐week GPE and GSE supplementation on them could still reflect the trend of recovery.

Figure 3c shows the heatmap of microbiota 16S rDNA analysis. It can be seen that GPE and GSE had a modest effect on gut microbiota restore. After treatment of antibiotic and restore, Gram‐negative bacterium generated greater changes compared to Gram‐positive bacterium. *Unclassified Enterobacteriaceae* (gram‐negative bacterium) had the highest proportion after recovery, with relative abundance of *Akkermansia* (gram‐negative bacterium) close behind. In contrast, GPE and GSE had no good for the restore of *Bifidobacterium* (gram‐positive bacterium), *Lactococcus* (gram‐positive bacterium), and *Lactobacillus* (gram‐positive bacterium). In another word, GPE and GSE had greater effects on the recovery of Gram‐negative bacterium than Gram‐positive bacterium.

## 4 DISCUSSION

The human distal gut is one of the most complex ecosystems on the planet. However, it may be a tractable and powerful system for the study of both basic ecological principles and health‐related community interactions through the exploitation of disturbance (Dethlefsen & Relman, 2011). The association between the health benefits of grape polyphenols, such as amelioration of cardiovascular and obesity risk factors, and changes in intestinal microbiota composition had profound implication for the relationship between diet and chronic disease (Kim et al., 2015). Interactions of gut microbiota with functional food components and nutraceuticals, like polyphenols, and the impact of gut microbiota on human health have already been studied (Laparra & Sanz, 2010). Therapeutic potential of gut microbiota has caused wide attention (Smits, Bouter, De Vos, Borody, & Nieuwdorp, 2013). Therefore, the present study provided further evidence for the potential role of grape polyphenols in the regulation of gut microbiota thus may suggest the role of grape polyphenol for the regulation of host health.

Antibiotic treatment had tremendous impact on the overall taxonomic composition of gut microbiota. Even with short‐term administration of antibiotics may shift the microbiota to a long‐term state of dysbiosis (Lange et al., 2016). According to the study conducted by Vrieze et al. (2014), antibiotic reduced the absolute number of gram‐positive bacteria, with a compensatory increase in gram‐negative bacteria. To specify, different groups of intestinal bacteria that were reduced significantly by vancomycin belonged to the *Firmicutes* phylum (*Clostridium* cluster IV and XIVa, *Lactobacillus plantarum* and various butyrate‐producing species including *Faecalibacterium prausnitzii* and *Eubacterium hallii*), as well as known pathogens from the *Proteobacteria* phylum (*Escherichia coli*, *Haemophilus,* and *Serratia*) (Vrieze et al., 2014).

Relative abundance of gut microbiota was based on the number of pyrosequencing reads clustering into each refOTU after normalizing the number of reads per sample by using 16S rDNA genome sequencing. A 3‐week treatment of antibiotic cocktail can eliminate the most gut microbiota, for example, *Bacteroides*, *Clostridium,* and *Enterobacteraciae* (Rakoff‐Nahoum, Paglino, Eslami‐Varzaneh, Edberg, & Medzhitov, 2004; Wang et al., 2012) and can be recolonized with normal gut microbiota after withdrawal of antibiotics (Suárez‐Zamorano et al., 2015). In the current study, the gut microbiota, which was composed of diverse populations of commensal bacterial species, were mostly removed by a 3‐week antibiotic cocktail treatment with a significant loss of diversity and certain important taxa, such as *Akkermansia*, *Alloprevptella,* and *Prevotella* and then uncompletely recovered after withdrawal of antibiotics for 7d. This consisted of previous investigations.

The effect of ciprofloxacin on the gut microbiota was profound and rapid, with a loss of diversity and a shift in community composition occurring within 3–4 days of drug initiation, and communities began to return to their initial state by 1 week after the end of each course, but the return was often incomplete (Dethlefsen & Relman, 2011). After withdrawal of antibiotics, Abx‐treated mice were repopulated with microbiota immediately from their conventional former littermates by co‐housing them for 7 days (Suárez‐Zamorano et al., 2015). In the present study, gut microbiota was mainly composed of the phyla *Firmicutes*, *Bacteroidetes*, *Actinobacteria,* and *Proteobacteria*, and the *Verrucomicrobia* phylum was occasionally observed. While the post‐antibiotic dysbiosis was characterized by a significantly reduced diversity of the phyla *Firmicutes*, *Bacteroidetes,* and *Actinobacteria* together, with a markedly increase of the family *Enterobacteriacea* of *Proteobacteria*, in accordance with previous study (Lange et al., 2016).

It has been reported that grape polyphenols have physiological effect on human health and some of which is closely linked to modulation of gut microbiota (Kim et al., 2015). The bioavailability and effects of polyphenols also greatly depended on their transformation by specific components of the gut microbiota; meanwhile, polyphenols and their metabolites may also inhibit or stimulate the growth of specific bacteria, exert prebiotic‐like effects, modify the composition or activity of the gut microbiota, thus affect the intestinal ecology (Kim et al., 2015; Laparra & Sanz, 2010).

In our work, supplementation of GPE and GSE after the withdrawing of antibiotics did had some positive effects on the recovery of gut microbiota. The richness and diversity of microbiota in feces of GPE and GSE administration animals were increased compared to the HFD + Abx^+/−^ group. GPE and GSE supplementation during the resurrecting process of gut microbiota improved the composition and total number of gut microbiota. Relative abundance of *Verrucomicrobia* was significantly increased by GPE and GSE administration. A study conducted by Zhang et al. (2018) consisted with our result. The *Verrucomicrobia* phylum had a phylogenetically close relationship with *Chlamydiae* and *Planctomycetes* and was mainly made up by environmental microorganisms. In this phylum, *Akkermansia* was an attractive bacterium that was first isolated from human feces (Dubourg et al., 2013). While relative abundance of *Actinobacteria* was markedly lowered by GPE and GSE administration compared to HFD + Abx^+/−^ group, consisting with a previous research (Jiao et al., 2018).

After further analysis, we found that relative abundance of *Akkermansia* in feces was greatly recovered by GPE and GSE supplementation compared to the HFD + Abx^+/−^ group. This was in accordance with a previous study (Anhê et al., 2017). It has been reported that *Akkermansia*, a Gram‐negative mucin degrading bacterium, which accounts for 1%–5% of total gut microbiota in health mammals. Hubert et al. have observed that the abundance of *Akkermansia* decreased during obesity and diabetes compared with healthy people, and that higher baseline abundance was significantly associated with the improvement of cardiometabolic parameters in individuals with obesity undergoing caloric restriction (Plovier et al., 2016). And feeding of *Akkermansia* enhanced mucus thickness, intestinal endocannabinoid production, and gut barrier function in mice on HF diets, which resulted in reduced fat mass, endotoxemia, adipose tissue inflammation, and insulin resistance (Everard et al., 2013; Kim et al., 2015; Shin et al., 2014), suggesting that GPE and GSE may ameliorate obesity and diabetes partly by increasing the relative abundance of *Akkermansia* in the gut. Moreover, since *Akkemansia* belongs to *Verrucomicrobia*, the improved *Akkemansia* was the major contributor to the observed increase of the relative abundance of *Verrucomicrobia* in present investigation.

## 5 CONCLUSION

Our findings suggested a light modulation of gut microbiota by grape pomace polyphenols on the restore of gut microbiota. The gut microbiota was characterized by a significant loss of diversity and certain important taxa after a 3‐week antibiotic cocktail treatment. Compared to the HFD + Abx^+/−^ group, supplementation of GPE and GSE during the recovery period of gut microbiota had some positive effects on relative abundance and diversity of microbiota in feces. Especially relative abundance of *Akkermansia* in feces was greatly elevated by GPE and GSE intake. This study also suggested that pharmacological or nutritional modulation of gut microbiota was an effective therapeutic method for the intestinal disorders making by antibiotics.

## CONFLICT OF INTEREST

The authors notify that there are no conflicts of interest.

## ETHICAL APPROVAL

This study does not involve any human or animal testing.
